# Supplementary material for: Development and Validation of a Risk Score for Post-Transplant Lymphoproliferative Disorders among Solid Organ Transplant Recipients
Source: Cancers (Basel). 2022 Jul 4;14(13):3279. doi: 10.3390/cancers14133279 (PMC9265532; doi:10.3390/cancers14133279)
Supplement: Supplementary file 1 [file cancers-14-03279-s001.zip › Supplemental material Figure S1.pdf]

A)

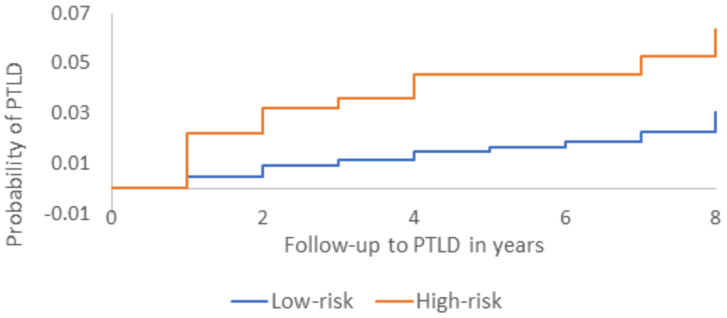

B)

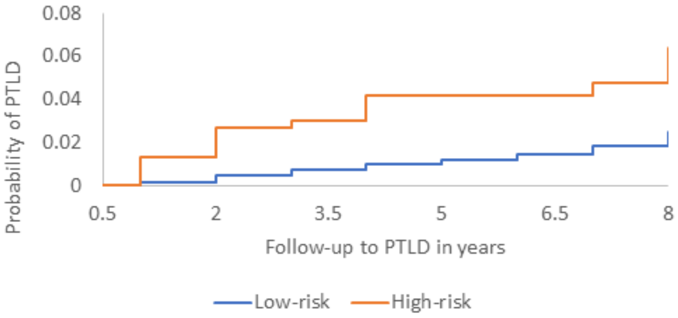

C)

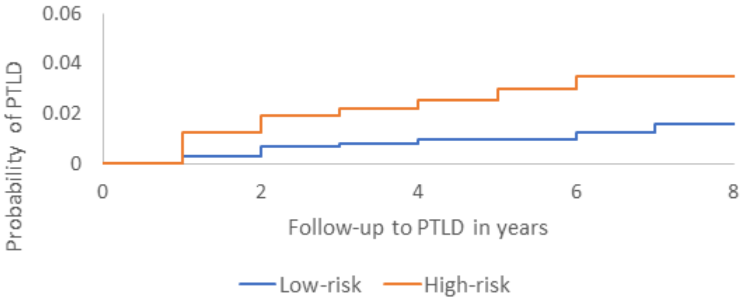

Number of patients at risk				
	MATCH		STCS	
Time (years)	Low risk	High-risk	Low risk	High-risk
0	2122	424	1084	445
2.0	1498	260	868	320
4.0	1107	187	611	214
6.0	808	138	407	130
8.0	570	84	198	66

D)

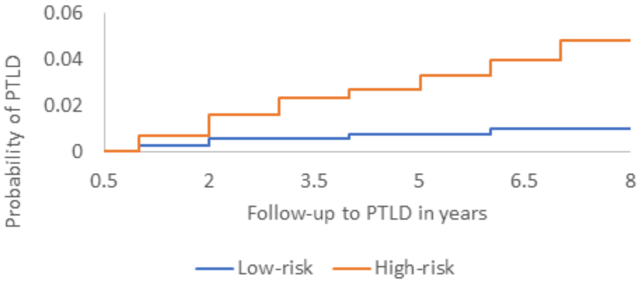

Number of patients at risk				
	MATCH		STCS	
Time (years)	Low risk	High-risk	Low risk	High-risk
0.5	1787	469	1083	443
2.0	1433	325	868	320
3.5	1137	248	667	237
5.0	908	197	497	165
6.5	727	146	358	111
8.0	541	113	198	66
