# Supplementary material for: Development and Validation of a Risk Score for Post-Transplant Lymphoproliferative Disorders among Solid Organ Transplant Recipients
Source: Cancers (Basel). 2022 Jul 4;14(13):3279. doi: 10.3390/cancers14133279 (PMC9265532; doi:10.3390/cancers14133279)
Supplement: Supplementary file 1 [file cancers-14-03279-s001.zip › Supplemental material File S1.pdf]

Liver transplantation

Following liver transplantation, a single dose of methylprednisolone 1,000 mg is given intraoperatively. In the days following the transplantation, prednisolone is tapered gradually from 200 mg on day 1 to 30 mg on day 5. For the remaining first month, 20 mg is given daily tapered to 15, 10 and 7.5 mg daily until month 6. For the next 6 months, 5 mg is given daily after which the drug is discontinued. Tacrolimus is given twice daily, aiming at through levels of 10–12 ng/mL in the first month, 8–10 ng/mL in month 2, 7–9 ng/mL in months 3–6, 6–8 ng/mL in months 7–12 and 4–6 ng/mL after 1 year. Mycophenolate mofetil is given twice daily at a dosage of 1,000 mg continuously. In addition, liver-kidney transplant recipients are given a dose of basiliximab 20 mg on day 0 and 4.

Heart transplantation

Following heart transplantation, thymoglobulin 1.5 mg/kg for 3 days with 1 g methylprednisolone for 2 days followed by 125 mg for an additional 3 days is used. Prednisone 0.2 mg/kg, tapered to 0.1 mg/kg after 3 months and to zero after 1 year, and mycophenolate mofetil 1–1.5 g × 2 daily continuously. Cyclosporine (or tacrolimus) is used adjusted to through blood levels between 200 and 350 ng/mL (10-15 ng/mL) for the first 6 weeks then reduced to 150–250 ng/mL (8-12 ng/mL) and again to 100 ng/mL (5-8 ng/mL) after one year.

Lung transplantation

Following lung transplantation, thymoglobulin 1.5 mg/kg for 3 days with 500 mg methylprednisolone for 1 day followed by 125 mg methylprednisolone each day for 3 days is used. Prednisone 15 mg daily tapered to 5 mg daily over 4 weeks. Azathioprine 1.5 mg/kg from postoperative day 1. Cyclosporine (or tacrolimus) from postoperative day 1 with target range 200–240 ng/mL (10-15 ng/mL) for the first 3 months, 150–200 ng/ml (8-12 ng/mL) for months 4–12, and again to 100-150 ng/mL (5-8 ng/mL) after one year.

Kidney transplantation

Following kidney transplantation, typically, induction is given with two doses of 20 mg basiliximab and methylprednisolone 250 mg immediately before transplantation. Maintenance immunosuppression is started at day 1 as triple therapy with Tacrolimus (target range 5-10 ng/ml), mycophenolate mofetil (750 mg x 2) and prednisolone (20 mg daily tapered to 5 mg daily the following month).
